# A Case of Clozapine-Induced Myocarditis in a Young Patient with Bipolar Disorder

**DOI:** 10.1155/2015/283156

**Published:** 2015-08-30

**Authors:** Ronny Cohen, Alla Lysenko, Thierry Mallet, Brooks Mirrer, Michael Gale, Pablo Loarte, Robert McCue

**Affiliations:** ^1^Chief of Cardiology, Woodhull Medical Center, 760 Broadway, Brooklyn, NY 11206, USA; ^2^Pharmacy Department, Woodhull Medical Center, 760 Broadway, Brooklyn, NY 11206, USA; ^3^Department of Medicine, Woodhull Medical Center, 760 Broadway, Brooklyn, NY 11206, USA; ^4^Division of Cardiology, Woodhull Medical Center, 760 Broadway, Brooklyn, NY 11206, USA; ^5^Department of Internal Medicine, Yale-New Haven Hospital, 20 York Street, New Haven, CT 06510, USA; ^6^Woodhull Medical Center, 760 Broadway, Brooklyn, NY 11206, USA

## Abstract

We present a case of drug-induced myocarditis manifesting as acute heart failure in a young patient with bipolar disorder being treated for depression. The case describes a 20-year-old man being treated in the psychiatry ward for worsening depression when he started complaining of chest pain and shortness of breath. His list of medications included clozapine, lithium, lorazepam, and haloperidol. The main findings on physical examination were tachycardia, low-grade fever, crackles in both lung bases on auscultation, and the absence of any notable edema. Abnormal labs included a troponin of 0.9, with a CK of 245 and CK-MB of 3.1. An ECG revealed sinus tachycardia and left anterior fascicular block (LAFB). An echocardiogram revealed global hypokinesis, severe left ventricular dysfunction with an ejection fraction estimated at 20%. The patient had an admitting diagnosis of acute left ventricular systolic dysfunction likely secondary to drug-induced myocarditis (suspect clozapine) versus acute coronary syndrome. He was managed conservatively and transferred to another facility for endomyocardial biopsy confirming myocarditis. This case is an example of one of the most typical presentations of suspected drug-induced acute myocarditis and will hopefully prompt the reader to think of this underdiagnosed entity in the right clinical setting.

## 1. Introduction

Myocarditis is an inflammatory disease of the myocardium that may lead to serious complications. The condition presents with a wide range of symptoms, some of which are remarkably nonspecific [1], while others point in the direction of myocyte injury. One of the more typical presentations of myocarditis is acute heart failure related to nonischemic severe systolic dysfunction in patients between 20 and 50 years old, with progression to dilated cardiomyopathy. The symptomatology is highly dependent on the causality and severity of the inflammation. The most common etiology is viral; others include bacterial, fungal, drug-induced, and hypereosinophilic syndrome [2]. In general, patients with fulminant myocarditis tend to be sicker but also recuperate more quickly with a good prognosis. However, patients with the chronic form of myocarditis can slowly develop their symptoms within weeks or months but will tend to develop a lingering disease requiring prolonged treatment and sometimes leading to end-stage CHF dilated cardiomyopathy requiring cardiac transplantation [3].

## 2. Case Presentation

A 20-year-old Hispanic male with past medical history significant for obesity (Body Mass Index 34), abnormal LFTs, and bipolar disorder was admitted to the psychiatric ward for worsening depression likely related to noncompliance. His home medications included benztropine, lithium, zolpidem, and chlorpromazine. On admission, the patient had a trial of aripiprazole and haloperidol without any significant improvement. Because of the patient's deteriorating psychiatric condition and the ineffectiveness of the previous antipsychotics, the decision was made to start the patient on clozapine 25 mg daily. The clozapine dosage was progressively titrated up over two weeks to a total daily dosage of 250 mg. On day 14 following the initiation of clozapine, the patient complained of chest pain. He described the chest pain as sharp, aching in nature, located in the center of his chest, and nonradiating, with an intensity of 7/10 on the pain scale. The chest pain was exacerbated with deep inspiration and lying supine. The patient also had mild shortness of breath, nausea, vomiting, dizziness, headache, palpitations, and nonproductive cough. The pain was not alleviated by any measures. The patient was transferred to the telemetry unit for close monitoring.

Vital signs were blood pressure of 103/61 mm Hg, heart rate of 100 bpm, respiratory rate of 19, and body temperature of 97.2 F with later maximal temperature of 101.8 F. Physical examination was unremarkable for icterus or redness or swelling in the oropharynx; the neck was supple; there was no JVD; auscultation of the heart revealed the presence of normal S1 and S2, with no additional heart sounds or murmurs; lungs were clear to auscultation bilaterally; the abdomen was soft, nontender, and nondistended; there was no edema of the extremities; and no rash was noted.

**Figure 1:**
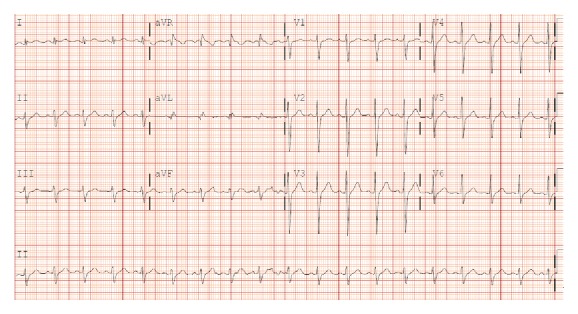
ECG sinus tachycardia. Left axis deviation late precordial lead transition.

Laboratory evaluation revealed a BNP of 121 pg/mL (normal < 100 pg/mL); initial troponin was 0.949 [nl] with peak of 0.976 and troponin of 0.333 was noted more than 24 hours later. Total CK was 245 and CK-MB was 3.1, [nl]. Cardio-CRP of 58.2, [nl], was suggestive of infection or inflammation. Basic chemistries were normal; CBC was unremarkable except for a slightly elevated eosinophil count. Urine drug screen and hepatitides B and C were all negative. Blood cultures showed no growth. Ferritin, ceruloplasmin, and alpha-1-antitrypsin were all normal. The electrocardiogram (Figure 1) showed sinus tachycardia, left axis deviation with left anterior fascicular block, and QTC prolongation (486 ms).

Transthoracic echocardiogram revealed four-chamber dilatation, biventricular failure, left ventricular apical akinesis, global hypokinesis, concentric LV hypertrophy, severely depressed LV systolic function with an ejection fraction of 20%, mild mitral and tricuspid regurgitation, and estimated pulmonary artery systolic pressure of 35 mm Hg.

As Acute Coronary Syndrome was a less likely possibility, the diagnosis of myocarditis presenting as nonischemic dilated cardiomyopathy was strongly considered. Clozapine was held due to this high suspicion and its association with drug-induced myocarditis. The patient was managed with furosemide, ramipril, and carvedilol; the doses of the medications were adjusted based on the patient's clinical response. Nonsteroidal anti-inflammatory drugs and acetaminophen were not given due to the history of abnormal liver function tests and given the acute phase of myocarditis with risk of possible viral agent propagation. Slight clinical improvement was observed, and the patient was ultimately transferred to another facility for endomyocardial biopsy confirming the diagnosis of myocarditis. He eventually did well with supportive treatment and was discharged home.

## 3. Discussion

### 3.1. Definition and Clinical Features

Myocarditis is an inflammatory disease of the myocardium. The initial criteria for the definition of myocarditis were based on the standard Dallas pathological criteria and required an inflammatory cellular infiltrate with or without associated myocyte necrosis to be present on conventionally stained heart tissue sections [4]. However, because of limitations in the interpretation and prognostic value, new criteria that rely on cell-specific immunoperoxidase stains for surface antigens, such as anti-CD3, anti-CD4, anti-CD20, anti-CD8, and anti-HLA, were developed [5]. Both criteria may be used for the diagnosis.

Acute myocarditis is frequently first diagnosed as nonischemic cardiomyopathy. Clinical manifestations are variable and can range from subclinical disease to sudden death [3]. Although a viral prodrome with fever, myalgia, and respiratory or gastrointestinal symptoms is classically associated with myocarditis, reported symptoms are highly variable [2, 3]. Cardiac symptoms are also variable and may include fatigue, decreased exercise tolerance, palpitations, precordial chest pain, syncope, dyspnea, and arrhythmias [3]. There seems to be a slight preponderance in male patients, which may be due to a protective effect of natural hormone variations on immune responses in women [3, 6].

Two main clinicopathological forms of acute myocarditis are generally described: fulminant lymphocytic myocarditis and acute lymphocytic myocarditis. The fulminant lymphocytic myocarditis has a distinct onset with a viral prodrome within two weeks before the onset of symptoms and hemodynamic compromise, but with generally a good prognosis. The acute lymphocytic myocarditis frequently does not have a distinct onset or hemodynamic compromise but more frequently results in death or the need for cardiac transplantation [3]. However, both forms of myocarditis are rare and prognostic data are only limited to relatively few patients.

Additionally, two chronic clinicopathological forms of myocarditis are recognized: chronic active myocarditis occurs with frequent clinical and histologic relapses, with ventricular systolic dysfunction associated with fibrosis and chronic inflammation on biopsy. The second one is chronic persistent myocarditis with biopsy showing persistent inflammatory infiltrate and small foci myonecrosis, with concomitant symptoms of chest pain or palpitation, despite normal and stable ventricular systolic function.

### 3.2. Causative Agents/Clozapine as a Cause of Myocarditis

Viruses remain the major causes of myocarditis. Historically, outbreaks of myocarditis have been linked to Coxsackievirus B from the 1950s through the 1990s and then to adenovirus in the late 1990s and more recently to parvovirus B19 [3, 7]. Other viruses, like hepatitis C, EBV, CMV, HHV-6, and HIV, have also been incriminated [3]. In addition to viruses, other infections, such as* Borrelia burgdorferi* and* Trypanosoma cruzi,* should also be considered. Drug-induced hypersensitivity reactions and systemic hypereosinophilic syndromes can also cause myocarditis. Among medications, anticonvulsants, antibiotics, and antipsychotics, as with clozapine, are the main culprits [3].

Myocarditis is one of the most publicized cardiac complications of clozapine treatment. Clozapine is a second-generation antipsychotic agent that has been shown to be effective in patients with schizophrenia and other psychotic disorders who have not responded adequately to other antipsychotic agents [8]. According to clinical research, clozapine is more effective than other antipsychotics and is associated with a significant decrease in suicidality in the treated patients [9–11]. Despite its efficacy, the general use of clozapine in clinical practice is limited because of the risk of several serious adverse effects such as agranulocytosis, neutropenia, and, rarely, cardiomyopathy.

Clozapine is the only antipsychotic known to cause myocarditis. Case series on clozapine-induced myocarditis by Kilian et al. [12] provided strong evidence that association was causal with the substantial incidence of 1 in 500 in the first month of clozapine use. More evidence came from subsequent publications by Coulter et al. [13], where the incidence was more than 2% of patients starting clozapine. Another study by Hägg et al. suggests that, in more than 85% of the cases, it typically occurs within the first 2 months and up to 75% within 3 weeks of treatment [14]. Nevertheless, infrequently, the symptoms of clozapine-induced myocarditis may develop more than two years into the treatment [15].

### 3.3. Pathogenesis

Although data is scarce on the pathophysiology of clozapine-induced myocarditis, there are a few proposed mechanisms. One hypothesis states that clozapine-induced myocarditis likely results from a type I Ig E-mediated acute hypersensitivity reaction. The time of onset of clozapine-induced myocarditis and peripheral eosinophilia along with eosinophilic myocardial infiltrates frequently observed in the course of disease all support this hypothesis [12, 16]. This has been seen in the observations of peripheral eosinophilia and eosinophilic inclusions within endomyocardial biopsy samples of affected patients, but findings are inconsistent. Other possible mechanisms of clozapine-induced myocarditis involve clozapine-induced cytokine release and hypercatecholaminemia [17]. When in vivo, it was noted that clozapine leads to release of tumor necrosis factor alpha and various interleukins. These proinflammatory cytokines have been found to mediate autoimmune myocarditis and may act similarly in clozapine-induced myocarditis [18]. Clozapine is known to increase serum catecholamine levels; cocaine has a similar effect and has been shown to exacerbate viral myocarditis, suggesting a role for catecholamines in the development of the disorder [19]. Elevated levels of norepinephrine have also been linked to the therapeutic efficacy of clozapine [12, 16]. It has been noted that patients treated with clozapine had higher noradrenaline levels than patients treated with other antipsychotics [17].

### 3.4. Diagnosis

A few diagnostic studies might guide us in making the diagnosis of myocarditis. Biomarkers of cardiac injury are elevated in a minority of patients with acute myocarditis but may help confirm the diagnosis [3]. Experimental data suggest that increased levels of troponin I are more common than increased levels of creatine kinase MB in acute myocarditis [20].

The electrocardiogram (EKG) may show sinus tachycardia with nonspecific ST-segment and T-wave abnormalities. Occasionally, the EKG changes might be suggestive of either an acute MI or pericarditis, with ST-segment elevation, ST-segment depression, or pathologic Q waves [3].

Echocardiography is useful primarily to rule out other causes of heart failure, since there are no specific features of acute myocarditis. Various echocardiographic patterns have been described. Wall motion abnormalities can simulate MI, but the loss of right ventricular function was the most powerful predictor of death or the need for cardiac transplantation in patients with biopsy-proven myocarditis [3, 21].

Cardiac MRI is being used with increasing frequency to evaluate suspected acute myocarditis, notably to localize suitable sites for an eventual endomyocardial biopsy [3, 22]. The latter should be performed in patients with unexplained, new-onset heart failure of less than two weeks' duration in association with a normal-size or dilated left ventricle and hemodynamic compromise for suspected fulminant myocarditis. Endomyocardial biopsy should also be performed if heart failure is associated with arrhythmias, high degree heart block, or failure to respond to usual care within one to two weeks [3]. It is preferable that those biopsies are performed in specialized centers with sufficient expertise in their interpretation.

### 3.5. Treatment

Patients presenting with myocarditis and acute dilated cardiomyopathy should be managed according to the latest 2013 Heart Failure Practice Guidelines of the American College of Cardiology, American Heart Association [23]. Most patients will improve with a standard heart failure regimen that includes ACE inhibitors or ARBs, beta blockers, and diuretics. For patients who fail to improve with optimal medical management, a role for mechanical circulatory support, such as Ventricular Assist Device, has been suggested as a bridge to transplantation or recovery [24]. Patients recovering from acute myocarditis should refrain from aerobic activity for a period of months, depending on the severity of left ventricular dysfunction and the extent of recovery. The use of NSAIDs was associated with increased mortality [3]. If a medication is thought to be the likely culprit, like clozapine in our case, it should be discontinued.

In patients with acute myocarditis, therapy for arrhythmias is supportive, since such arrhythmias usually resolve after the acute phase of the disease. Temporary pacemakers may be required for patients with symptomatic bradycardia or complete heart block [3].

The finding of viral genomes on endomyocardial biopsy has been used to guide treatment. However, because most patients with acute myocarditis are diagnosed weeks after viral infection, it is unlikely that antiviral therapy would be provided early enough to be of benefit in acute viral myocarditis. In contrast, interferon beta has been used successfully in patients with viral persistence in chronic, stable dilated cardiomyopathy, with viral clearance being achieved in all patients and with significant increase in the left ventricular function [3, 25]. Studies done to evaluate the effect of immunosuppression with Intravenous Immune Globulin (IVIG) did not reach similar conclusions, and therefore the use of IVIG is not recommended [26]. More investigations are being done to evaluate if changes in immune regulation might be beneficial, particularly in patients with more chronic cardiomyopathy.

## 4. Conclusion

Our case presentation represents, most likely, drug-induced myocarditis with clozapine as the likely culprit. The clinical manifestations leading to the diagnosis were mainly chest pain and shortness of breath, but also confounding nonspecific symptoms. Diagnostic findings were suggestive of an inflammatory process affecting the myocardium, with elevated cardiac biomarkers, elevated CRP, sinus tachycardia, and dilated cardiomyopathy. Clozapine was discontinued and the patient was appropriately treated with ACE inhibitors, beta blockers, and diuretics, which resulted in clinical improvement. The patient was transferred to a specialized center where he had an endomyocardial biopsy, which confirmed the diagnosis of myocarditis. The roles of cardiac MRI and endomyocardial biopsy in the diagnosis were reviewed. New diagnostic modalities are being sought to try to reduce the role of endomyocardial biopsy, as it is an invasive measure. Most of the treatment is supportive, but new therapeutic modalities, particularly in terms of antiviral and immunomodulatory therapies, are being investigated.
